# A smartphone-based self-care application for patients with urinary tract stones: identification of information content and functional capabilities

**DOI:** 10.1186/s12894-022-01127-z

**Published:** 2022-11-15

**Authors:** Leila Shahmoradi, Amin Azizpour, Mahmud Bejani, Pejman Shadpour, Sorayya Rezayi, Jebraeil Farzi, Alireza Amanollahi

**Affiliations:** 1 Health Information Management and Medical Informatics Department, School of Allied Medical Sciences, Tehran University of Medical Sciences, Tehran, Iran; 2 Iranian Social Security Organization, Tabriz, Iran; 3 Health Information Management Department, School of Management and Medical Informatics, Tabriz University of Medical Sciences, Tabriz, Iran; 4 Hospital Management Research Center (HMRC), Hasheminejad Kidney Center (HKC), Iran University of Medical Sciences, Tehran, Iran; 5 Health Information Technology Department, School of Allied Medical Sciences, Zabol University of Medical Sciences, Zabol, Sistan and Balouchestan Iran; 6 Department of Epidemiology, School of Public Health and Safety, Shahid Beheshti University of Medical Sciences, Tehran, Iran

**Keywords:** Information elements, Functional capabilities, Self-care, Urinary tract stones, Application

## Abstract

**Purpose:**

This study aimed to identify and validate the information content and functional capabilities of a smartphone-based application for the self-care of patients with urinary tract stones.

**Methods and materials:**

First, by reviewing studies and urology-oriented books, studying 214 medical records, and consulting with specialists, the information items and basic capabilities of the application were identified, and in the next stage, a researcher-made questionnaire was designed based on the information obtained from the previous step. Then, experts' opinions were considered to confirm the validity and reliability of the questionnaire; the designed questionnaire was distributed among various participants. Finally, the application's leading information elements, contents, and functional capabilities were explored by analyzing the questionnaire results.

**Results:**

To conduct the survey, 101 patients with Urinary Stone Diseases (USD), 32 urologists and nephrologists, 11 nurses, and six other specialists were recruited. After analyzing the results of the filled questionnaire, 21 information elements and nine surveyed capabilities that were more important than others were selected to be used in designing the application. Some of the principal information elements that were used in the application design include: the cause of various stones in the body, clinical manifestations, laboratory results, treatments of various stones, the role of environmental factors in the treatment, the role of nutrition in the treatment and formation of stones, and different diagnostic methods. Some of the important features of the application include: medication and fluid intake reminders, laboratory test reminders, radiography and periodic examination reminders, surgical history, and easy access to medical centers for information. The mean score of information elements was 75.07 from the patients' perspective, 65.09 from the physicians' perspective, and 80.09 from the nurses' perspective. Also, the mean score of application capabilities was 31.89 from the patients' perspective, 30.37 from the physicians' perspective, and 35.09 from the nurses' perspective. The difference in the mean scores of the above variables was statistically significant (*p* < 0.05) in both layers.

**Conclusion:**

In this study, informational and functional needs and capabilities were presented for designing a mobile-based application that helps in disease management in patients with urinary tract stones.

## Introduction

Chronic diseases are highly significant and have a high priority for health care systems because they are challenging to manage, costly, and in most cases, lead to major disabilities [1]. Urinary tract stones or Urinary Stone Diseases (USD) are one of the most common, influential, recurrent painful disorders and chronic diseases with high prevalence due to modern lifestyle, poor nutrition, bad habits in fluid intake, and improper use of drugs [2, 3]; unfortunately, there is no doubt that USDs remain a significant health problem [4]. Small stones often pass through the body with little discomfort, but larger stones can be painful and even block the urinary tract [5].

Self-care means empowering people to maintain health and perform self-care activities, so that they would be able to look after their health and, in case of illness, take timely and correct measures to prevent disease complications and achieve faster recovery [6]. In many cases, self-care can also be effective in controlling and reducing disease complications, lowering treatment costs, and facilitating faster recovery [7]. Self-care interventions ultimately lead to a decrease in disease activity and its consequences, improve quality of life, general health, functional capabilities, and patient satisfaction, increase the use of health services, and also decrease the costs of the health system [8].

Managing chronic diseases (like kidney and urinary tract stones) is a complex process that typically requires individuals to manage a number of health-related factors themselves; some diseases require near-total self-care. Supporting self-care enables patients to self-identify problems and provides techniques to help them make decisions, act, and alter behaviors [4, 9]. Self-care skills are the capacity of an individual with a chronic disease to participate in a daily, self-motivated, collaborative (conducted with family, social, and healthcare provider support) process to manage symptoms. This process involves the domains of focusing on illness needs, activating resources, and living with a chronic illness. In chronic conditions, an individual’s ability to perform behaviors that will alleviate the pain experience is instrumental in adapting to prolonged-term pain [10].

Despite the need for proper management of chronic diseases and the role of patient awareness in this process, patients with urinary tract stones or USDs must have the necessary skills to self-care for their disease [11]. Management strategies for patients with urinary tract stones vary from surgery and stone removal with extracorporeal lithotripsy or transurethral lithotomy to complete control of the disease and management of stone reformation [12].

### Significance of the study

The annual incidence of USD in industrialized countries is reported to be 1500–2000 cases per one million [13]. The prevalence of USD is reported to be 1–5% in Asian countries, 5–9% in European countries, and 12% and 13–15% in Canada and the United States, respectively [14]. The prevalence of USD in Iran is 5.7% (6.1% in men and 5.3% in women) [15]. Notably, Iran has the highest percentage of USD among West Asian countries after Saudi Arabia and Turkey [16]. The nature of modern lifestyle, improper nutrition, bad habits in drinking liquids, and incorrect use of medicines are the reasons for the high prevalence of USD [17].

Communication and telecommunication technology development has revolutionized the world in recent years [18]. One of the most important applications of information technology is in the field of health and treatment, which promotes health, prevention, treatment, prognosis, and self-care [19, 20]. Thus, the integration of intelligent technologies and wireless communications, and mobile computing leads to the effective delivery of care, providing unique diagnostic services to individuals and managing chronic diseases [9]. Consequently, due to the high prevalence and incidence of USD, the development of self-care tools, especially mobile-based or digital self-care applications, is significant.

### Aim of the study

The main aim of this study was to determine and validate the information elements, content, and functional capabilities of a smartphone-based application to facilitate self-care in patients with urinary tract stones and prevent this disease.

## Methods and materials

This study was conducted in Shahid Hasheminejad Hospital, affiliated to Iran University of Medical Sciences in Tehran. This hospital is the main center for USDs in the country. This study is generally done in two phases:

### Extracting primary information elements based on a review of the texts and scrutinizing medical records

First, primary information elements for self-care and prevention of stone formation and reformation were identified and classified through a literature review, including urology reference books like Campbell Walsh Wein Urology book, comprehensive book of urology in Iran, European Association of Urology (EAU) guideline and scientific articles, scrutinizing records of 214 discharged patients and consulting with urology and health information technology experts. By using mesh terms, searches were conducted in the electronic databases of Medline (through PubMed), ISI Web of Science, Scopus, and Science Direct. Also, the UpToDate database, part of evidence-based medical databases, was searched. This comprehensive library survey was done in the period from 2000 to 2021. The keywords for searching scientific articles were: Urolithiasis, Nephrolithiasis, Kidney Stone, Renal Stone, Urinary Tract stone, Software, Medical app, and Urologic Medical App. At first, 101 articles were retrieved focusing on urinary tract stone disease and m-health and application design through advanced search in databases with the combination of keywords. During studying the titles and text of these searches, 58 articles were removed, and 107 papers were chosen. In addition to surveying related literature, clinical information, symptoms, and treatment information in patient records that could be used in the questionnaire design was selected under experts' guidance. Based on the literature review, experts' recommendations, and exploring patient records, essential and fundamental items in managing and self-care were identified and categorized, especially the critical information elements of self-care of USD.

### Identification and selection of final information elements and capabilities based on the survey

In the following, based on the previous step's results, an initial draft of a researcher-made questionnaire was developed. The final version of this questionnaire was designed by extracting different questions and items, combining them, and receiving various feedback from urology experts. The validity of this questionnaire was also reviewed and confirmed by a group of experts (two urologists, one nephrologist and three endourology fellowships) with Cronbach's alpha of 0.85. The questionnaire consists of 30 questions in three sections, including patient information, education and raising of awareness on the subject of urinary tract stones, and functional capabilities of the application. The study population consisted of patients with various urinary tract stones referred to Shahid Hasheminejad Hospital, urologists and nephrologists, nurses, and other medical staff working in Shahid Hasheminejad Hospital. The participants were selected from the target population using the convenience sampling method. The researchers visited the clinic and related departments of the hospital several times and provided the questionnaires to the patients, medical staff, and experts. Also, they provided the necessary explanations to the participants in this field and again visited in person and collected the questionnaire. Using the Delphi method, each of the information elements in the questionnaire was evaluated on a scoring scale of one to five based on the Likert scale, and each of them was considered as necessary item only if they obtained more than 70% of the relevant score (3 from 5). Information elements that obtained a score of less than 50% (less than 2.5 out of 5) were considered unnecessary and removed from the information elements. Also, in each section, an empty cell was considered so participants who completed the questionnaire could add another element to the section if they thought it necessary.

The survey questionnaire results were used to compare the mean scores of more than two layers, using analysis of variance, and also, Kruskal-Wallis and Mann-Whitney tests were used to compare the mean scores of two layers. Data were analyzed by SPSS software version 23 at a significant level of 0.05.

**Fig. 1:**
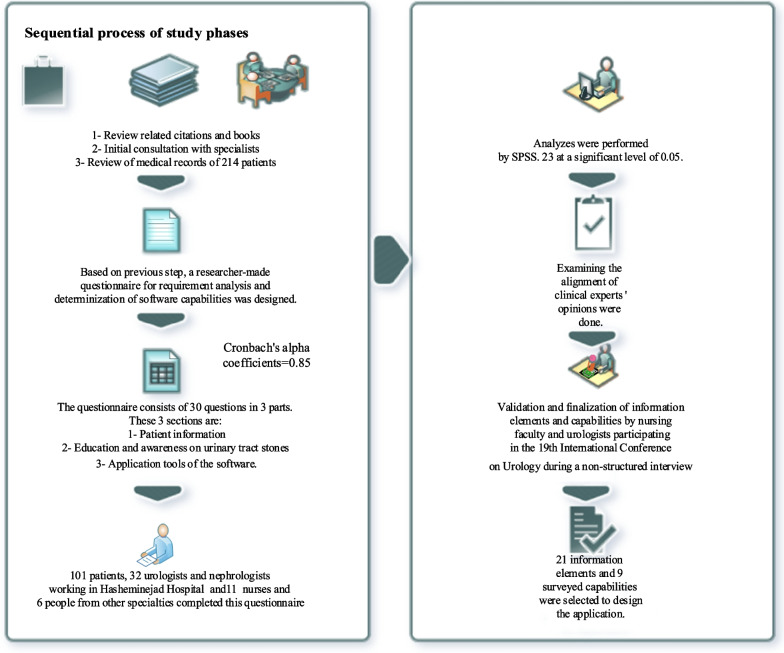
Main phases of the methodology

The information elements, content, and capabilities of the application extracted from the questionnaire were validated and finalized during a non-structured interview with nursing faculty members and urologists participating in the 19th International Conference on Urology. The main phases of the method are depicted in Fig. 1. Details of the application design and evaluation phase have been provided in the previous study by the authors [21].

## Results

### Descriptive information on participants

One hundred one patients, 32 urologists and nephrologists, 11 nurses, and six people from other specialties were selected to participate in the study. Of 101 study participants with urinary tract stones, 80 (79.2%) were men and 21 (20.8%) were women, and their maximum disease history was 30 years. Patients' weight was in the range of 52–110 kg, with a mean of 78.7 kg. Also, participants' height was in the range of 151–188 cm, with a mean of 172.34 cm.

**Fig. 2:**
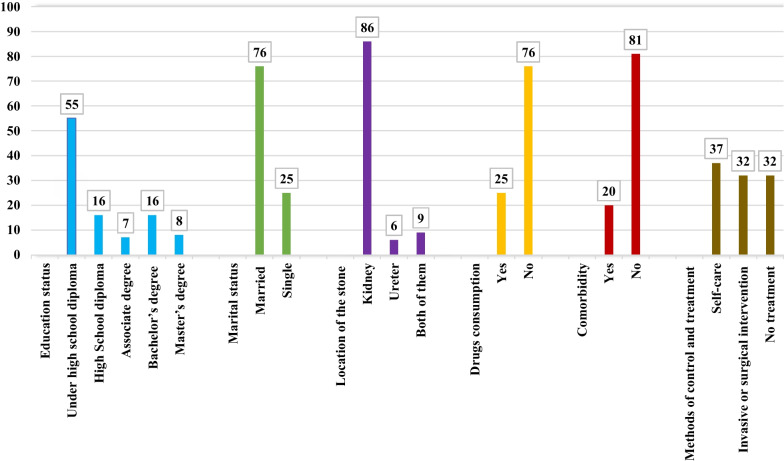
Education level, marital status, location of stones, medical history, comorbidities and methods of controlling and treating urinary tract stones

Figure 2 shows the patients' education level, marital status, location of stones, drug use, comorbidities, and methods of controlling and treating urinary tract stones.

**Fig. 3:**
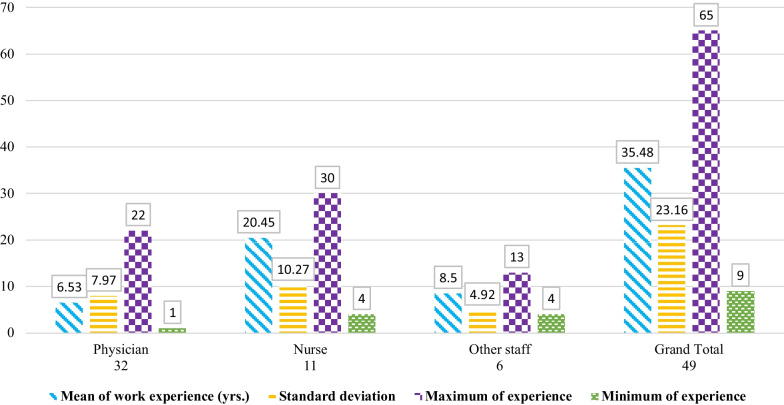
Descriptive information on physicians, nurses and other medical staff

Figure 3 shows the specialists’ information (physician, nurse, and other related medical staff). It also indicates the type of job, length of work experience by year, as well as the history of providing services to urological patients.

### Final information content and functional capabilities found by the questionnaire to design a self-care application

**Fig. 4:**
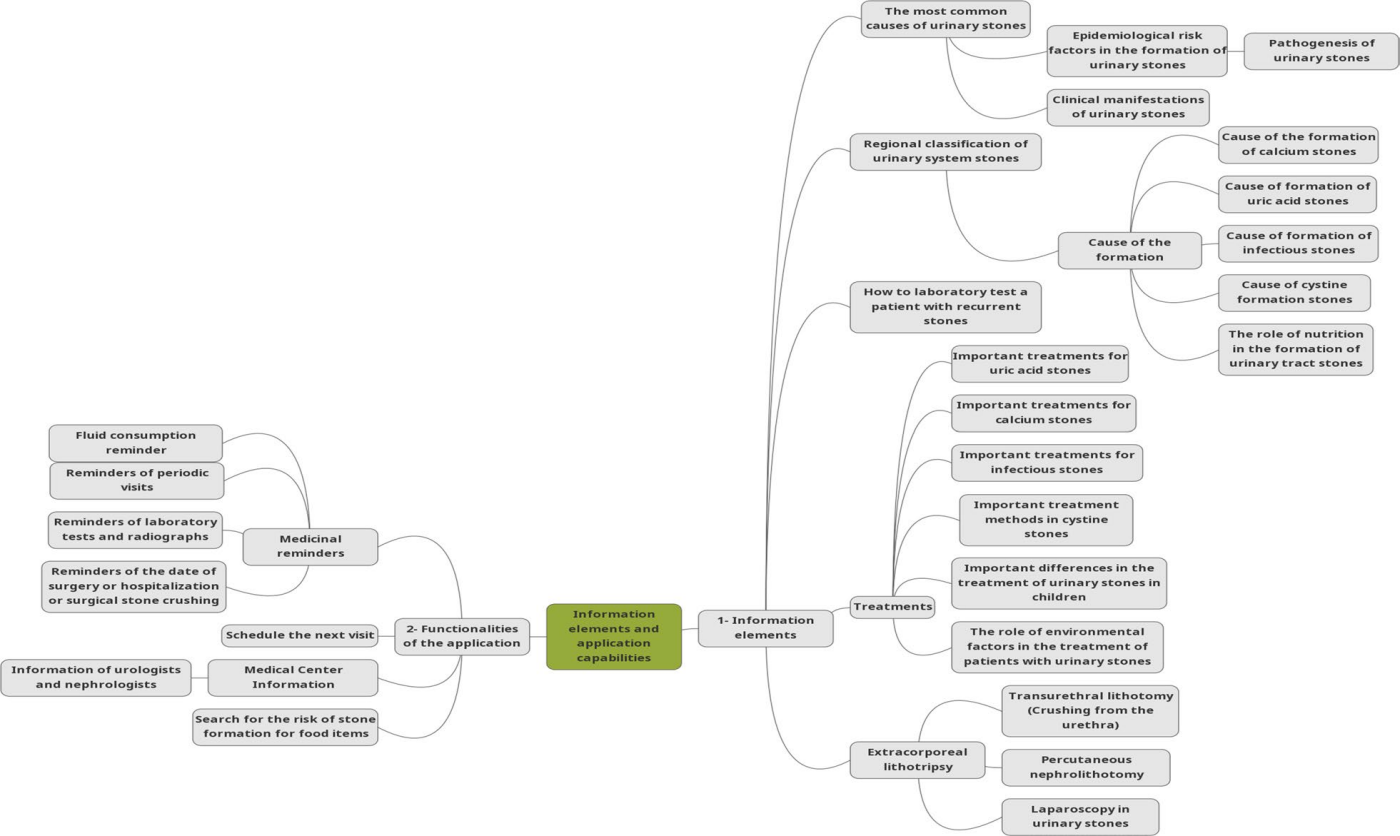
Chosen information content and functional capabilities

Figure 4 shows the chosen information items and capabilities for designing the self-care app based on participants' opinions (patients, specialists, nurses, and other medical staff). Finally, 21 information elements and nine surveyed capabilities that were more important than others were selected to be used in designing the application.

The questionnaire items are given in our previous article; information elements included in the final questionnaire are listed in a table. Also, the mean and standard deviation of scores for information elements and the application functionalities in the questionnaire is provided in this table [21].

Table 1 shows the mean scores of information needs assessment required for designing the self-care application for patients with USD according to the participants using the analysis of variance test. According to the table, the highest mean score of information elements related to the strata layer was 80.09 for nurses and 75.07 for patients. The difference in mean score between all three layers was significant (*p* < 0.001). Regarding the application capabilities, the highest score was related to the layer of nurses (35.09) and then the layers of patients (31.89) and physicians (30.37). The results showed a significant difference between the mean scores of the three layers (*p* = 0.005).

**Table 1:** The mean scores of information needs assessment and application capabilities required for the design of the app

Variable	Layer	Mean	SD	Highest	Lowest	*P*-value
Information elements (21 information elements)	Patient	75.07	6.51	84	57	*P* < 0.001
	Physician	65.09	8.24	79	53	
	Nurse	80.09	5.46	84	70	
	Others	64.0	3.28	67	61	
	Total	72.87	8.26	84	53	
Application capabilities (nine capabilities)	Patient	31.89	4.50	36	22	*P* = 0.005
	Physician	30.37	3.51	34	24	
	Nurse	35.09	1.3	36	33	
	Others	29.0	3.28	32	26	
	Total	31.68	4.27	36	22	

Table 2 shows the mean scores of information needs assessment and application capabilities according to the stone location, treatment history, drug use, comorbidity, and the type of stone based on a survey of patients referred to the medical center. The mean difference between the three layers was compared using Kruskal-Wallis test, and the two layers were compared using Mann-Whitney test. The difference in the mean score of information elements was significant regarding stone location (*p* = 0.038) and type of stone (*p* = 0.03). In addition, the difference in the mean score of application capabilities in terms of treatment history (*p* = 0.001) and stone type (*p* = 0.003) was also significant. Among the studied covariates, only the mean score of stone type was significant both in terms of the information elements and application capabilities. A history of drug use and comorbidities did not significantly affect the mean score of information elements and application capabilities.

**Table 2:** Comparison of differences in the mean score of information needs assessment and application capabilities

	Layer	Information elements	*P*-value	Application capabilities	*P*-value
Location of stone	Kidney	75.05 ± 6.86	0.038	31.55 ± 4.78	0.28
	Urethra	71.0 ± 3.28		35.50 ± 1.09	
	Both	78.0 ± 0.09		33.0 ± 0.09	
Treatment history	Self-care	74.59 ± 5.56	0.48	27.59 ± 3.56	0.001
	Surgery	74.15 ± 6.67		33.96 ± 2.27	
	No treatment	76.1 ± 7.41		34.65 ± 3.39	
History of medication use	Yes	74.64 ± 7.66	0.69	33.56 ± 2.31	0.08
	No	75.22 ± 6.12		31.34 ± 4.91	
Comorbidity	Yes	74.00 ± 8.04	0.59	32.95 ± 2.81	0.55
	No	75.34 ± 6.1		31.62 ± 4.89	
Type of stone	Identified	74.4 ± 2.17	0.03	31.2 ± 2.44	0.003
	Unidentified	75.43 ± 7.89		32.34 ± 5.24	

## Discussion

Data analysis showed that creating a self-care application for patients with USD is different from the perspective of patients, physicians, nurses, and other medical staff, so the highest score of information elements required for the self-care application was obtained by nurses and patients. Even in terms of application capabilities, patients and nurses have given the highest score to information elements, which shows that nurses and patients had the most confidence in the information elements of this application. However, in some covariates, the mean score between the information elements and application capabilities differed from the patients' point of view, which was observed regarding stone location and treatment history. Based on the results, the highest mean score of information elements related to the strata layer was 80.09 for nurses and 75.07 for patients. In fact, nurses and patients examined the main information items and practical capabilities of the application (information content and functional capabilities) more carefully and were more involved in designing their opinions. Among the studied covariates, only the average score of stone type was significant, and having a history of drug use and comorbidities did not make significant differences; therefore, in designing, our focus was more on information about the type of formation of stones, their treatment, and diagnosis.

In a study of [22], a smartphone-base application was developed to educate patients with End-Stage Renal Disease (ESRD). According to the results, the use of people involved in the application development (subject matter and information technology specialists), and people who were active in education and training applications to promote health, were influential in ensuring that the self-care application worked efficiently. The smartphone-based learning has been instrumental in empowering patients with ESRD and helping them in self-care because it facilitates access to information. One of the interesting points in this regard is that, in line with our study, the above study was initially started with a survey and continued with the cooperation of subject matter experts and patients.

In another study by Amor-García et al. [23], the quality of patient-centered smartphone-based applications for managing kidney stones was comprehensively evaluated. After searching for resources, eligible applications were divided between fluid and food applications. In total, 31 applications (18 fluid applications and 13 food applications) were included in the study. The fluid applications outperformed food applications in all aspects of the Mobile App Rating Scale (MARS), and food applications performed poorer, most of which were limited to non-interactive information content. It can be concluded that they do not have enough time and energy to design food applications, especially when identifying information requirements and appropriate content. Whereas, in our study, the information elements extracted from the opinions of experts, medical staff, and patients were evaluated and validated to design a practical application to control and prevent USD. All information content and functional/non-functional capabilities of the self-care application were carefully and explicitly extracted in different phases and used in the design stage.

According to recent studies, each person (patient, physician, nurse, or designer of applications) should use the information sources that exist in the applications according to their needs and avoid using different applications. In this regard, there is also a discussion of information pollution among the available sources, which should be considered in developing applications with specialized items. More participation of physicians, clinicians, and treatment staff in the design of health-related applications should also be regarded. However, the accuracy of contents and specialization of designs are more considered currently, as studies in this field show that these issues should be taken more remarkably. This is while, in 39% of urology apps, the renal and urinary tract specialists have been surveyed to develop [24–26].

To accurately identify the requirements and information elements, we tried to maximize the participation and cooperation of the medical staff and experts in the field of USD treatment and research and also use the experiences and opinions of doctors, nurses, and other related specialists. Patients' clinical records and surveys were also used to extract patients' needs and make the app more useful and comprehensive for users.

Alexander Small and colleagues conducted a study entitled [27]: “Designing an emergency mobile platform for assisting and managing patients with kidney and urinary tract stones”. In line with our study, this smartphone-based application was designed for patients with kidney and urinary tract stones and aimed to enhance hydration, improve diet, reduce medication side effects and monitor the disease symptoms. The rate of non-adherence to treatment in patients with kidney and urinary tract stones is similar to other chronic patients, many of whom suffer from symptoms of lower urinary tract disease, recurrent infections, and chronic pain. Designed applications can increase treatment adherence, facilitate the prevention and reformation of urinary tract stones, and promote telemedicine. Hence, smartphone-based applications provide significant advantages for patients, healthcare providers, and researchers.

Valent and colleagues [28] developed a smartphone-based application for controlling kidney stone disease called “Hydriney”. In patients with urinary tract stone, who need daily water consumption, this application could be a tool to monitor daily water intake and urine PH. This application is able to meet the needs of patients and facilitate the management of urinary tract stones, and by using the reports provided by this software, urologists could better treat and manage the disease and increase the quality of life of patients.

One of the strengths of present study is the survey of specialists and patients at its initial stage to identify the information elements and functional capabilities of the application, which increase the generalization of our results. One of the significant limitations of this study is the lack of cooperation of some patients referred to the hospital.

## Conclusion

In the field of disease control, diagnosis, and patient management, education has an important role, as it helps patients to acquire self-care skills, improve their health and follow the treatment process. Some of the leading information elements included: the cause of various stones in the body, clinical manifestations, laboratory results, treatments of various stones, the role of environmental factors in the treatment, the role of nutrition in the treatment, and methods of stone formation and detection. Some of the critical capabilities of the app include: medication and fluid intake reminders, surgical history, easy access to medical centers for obtaining information, laboratory test reminders, and radiography and periodic examination reminders.

## Data Availability

All data generated or analyzed during this study are included in this published article.
